# Insomnia, Fatigue, and Depression: Theoretical and Clinical Implications of a Self-reinforcing Feedback Loop in Cancer

**DOI:** 10.2174/1745017902117010257

**Published:** 2021-12-31

**Authors:** Laura Palagini, Mario Miniati, Dieter Riemann, Luigi Zerbinati

**Affiliations:** 1 Department of Neuroscience and Rehabilitation, University of Ferrara, Via Fossato di Mortara 64/A, 44121, Ferrara, Italy; 2 Department of Clinical and Experimental Medicine, Psychiatric Clinic, University of Pisa, Via Roma 67, 56100, Pisa, Italy; 3 Department of Psychiatry and Psychotherapy, Medical Center, Faculty of Medicine, University of Freiburg, Hauptstraße, 579104, Freiburg, Germany

**Keywords:** Insomnia, Cancer, Depression, Cancer-related fatigue, Cognitive-behavioral therapy-insomnia, Stress

## Abstract

**Introduction:**

Insomnia is emerging as a modifiable major risk factor for mental and physical problems, including cancer, and it may contribute to cancer-related fatigue and depression. Since both fatigue and depression may favor insomnia as well, we may hypothesize a self-reinforcing feedback loop among these factors in cancer.

**Methods:**

With the aim of discussing this hypothesis, PubMed, PsycINFO, and Embase electronic databases were searched for literature published according to the PRISMA method with several combinations of terms such as “insomnia” and “cancer” and “fatigue” and “depression”. On this basis, we conducted a narrative review about theoretical aspects of insomnia in the context of cancer and about its role in cancer-related fatigue and depression.

**Results:**

Twenty-one papers were selected according to inclusion/exclusion criteria. Insomnia is frequent in cancer, and it is associated with cancer-related comorbid conditions such as emotional distress, depressive symptoms, and cancer-related fatigue. The hyperactivation of stress and inflammatory systems, which sustain insomnia, may contribute to cancer-related depression and fatigue. A deleterious feedback loop may be created, and it may perpetuate not only insomnia but also these cancer-related comorbid conditions.

**Conclusion:**

Although the understanding of the causal relationship between insomnia/ depression/fatigue in individuals with cancer is limited, we may hypothesize that these symptoms can exacerbate and maintain each other. When insomnia is established in cancer, it may lead to a vicious cycle with fatigue and depression and may contribute to adverse cancer outcomes. Interventions targeting insomnia could provide a promising approach not only for insomnia but also for cancer-related symptoms among cancer patients.

## 1 INTRODUCTION

Sleep serves important regulatory functions influencing mood, emotion regulation, impulse behavior, and stress adjustments [1, 2], while sleep problems are recognized as major risk factors for mental and physical problems, including cancer [3-6]. Disturbed sleep is frequent in patients with cancer, and it is rated the second most disturbing symptom in this population [7-9]. The frequency of sleep disturbances in oncologic patients is more than 2 times higher than in the general population and might affect from 25 to 75% of newly diagnosed patients, those undergoing active cancer treatment or those who completed cancer treatment [8-11]. Even if sleep of disorders can occur singly or in combination and include different disturbances, insomnia symptoms are the most frequent in the oncologic population affecting from 30 to 50% of the patients [8-11]. Consequences of insomnia in cancer patients include mood symptoms, psychological distress, daytime fatigue, increased pain, increased chances of cancer recurrence, medication misuse and abuse, impaired cognitive functioning, and quality of life [8-13]. Although insomnia is common throughout the cancer trajectory, it continues to be under-diagnosed and under-treated in clinical practice [12]; the burden of insomnia in patients with cancer still needs to be addressed [8-12]. Since depression and cancer-related fatigue can also negatively affect sleep quality and duration in cancer [11], we may hypothesize a self-reinforcing feedback loop among these factors. By treating insomnia, we may be able to interrupt their vicious cycle. With the aim of discussing this hypothesis, we conducted a theoretical overview on the association among insomnia, fatigue, and depression in cancer. A revision of theoretical aspects of insomnia, cancer-related fatigue, and depression in cancer has been performed, and clinical and therapeutic implications have been discussed under the form of a narrative review.

## 2 METHODS

The PubMed, PsycINFO, and Embase electronic databases were searched for literature published according to the PRISMA (Preferred Reporting Items for Systematic reviews and Meta-Analysis) method. Several combinations of search terms were used, such as “insomnia,” “cancer,” “fatigue,” and “depression,” Inclusion criteria were 1) interested patients with cancer 2) full text available in English, 3) performed up to May 2021. Systematic reviews and meta-analyses were included, while papers were excluded if they concerned other sleep disorders such for example sleep apnea or restless leg syndrome. On this basis, we conducted a narrative review about theoretical aspects of insomnia in the context of cancer and about its role in cancer-related fatigue and depression.

## 3 RESULTS

Twenty-one papers were selected according to inclusion/exclusion criteria. Insomnia is frequent in cancer, and it is associated with cancer-related comorbid conditions such as emotional distress, depressive symptoms, and cancer-related fatigue. In part 1) Insomnia and cancer, we discussed models of insomnia and reviewed clinical and therapeutic implications of insomnia treatments in cancer. In part 2) Insomnia and Cancer-Related Fatigue, we discussed the role of insomnia in cancer-related fatigue, and in part 3) Insomnia and depression in cancer, we discussed the role of insomnia in cancer-related depression. In part 4), we discussed theoretical hypotheses of a self-reinforcing feedback loop among insomnia, depression, and fatigue in cancer and clinical and therapeutic implications of their relationship.

## 4 DISCUSSION

Chronic insomnia, also currently referred to as “insomnia disorder”, now has similar diagnostic criteria in the American Psychiatric Association's *Diagnostic and Statistical Manual of Mental Disorders, Fifth Edition* (DSM-5) (APA 2013) [14] and in other sleep manuals. Insomnia disorder is now considered a 24-hour *sleep*-wake *disorder* [14] characterized by nocturnal and diurnal symptoms. Insomnia can be episodic, lasting for a period within 1 month or between 1 month-3-months, or persistent, lasting longer than 3 months; transient-episodic forms tend, in the majority of the cases, to chronicity.

Insomnia is defined as difficulty initiating or maintaining sleep, early-morning awakening, or non-restorative sleep associated with daytime consequences such as fatigue, irritability, and lack of concentration [14]. It is the most frequent sleep disturbance interesting almost one third of the general population. Frequency, severity, and pattern of insomnia can vary in cancer, with insomnia affecting more than 50% of patients with lung and breast cancer, almost 40% of patients with other forms of cancer, and at the first cycle of chemotherapy or during the peri-operative period.

The evolving models of chronic insomnia according to neurobiological, neurophysiological, cognitive, behavioral, or other perspectives [15] made the evaluation of insomnia progressively more complex. Although details of current models are beyond the scope of this paper, concepts are critical for insomnia evaluation in cancer. The most heuristic model of insomnia is the diathesis-stress model proposed, commonly known as the “3-P” model, describing Predisposing, Precipitating, and Perpetuating factors relevant to the development and maintenance of insomnia [15]. *Predisposing factors* include genetic, physiological, or psychological diatheses that confer differential susceptibility to individuals in response to stress. *Precipitating factors* include physiological, environmental, or psychological stressors interacting with predisposing factors to produce acute symptoms. *Perpetuating factors*, especially behavioral, cognitive, and environmental factors, intervene in the perpetuation of insomnia.

Multiple factors may contribute to the development and maintenance of insomnia in oncologic patients. These may include fatigue and depression, tumor pathology, advanced stage of cancer, cancer-related treatments, adjunct medications, environmental factors, psychosocial disturbances, and comorbid medical conditions [8-11, 16, 17].

*Predisposing factors* in oncologic patients include advanced age, female sex, an anxiety-prone personality, a family or personal history of insomnia and/or psychiatric disorder, pain and vasomotor symptoms, daytime fatigue, and irregular exposition to light [16, 17]. Oncologic patients are exposed to a myriad of *precipitating factors* [16, 17]. These include anxiety/distress related to the cancer diagnosis, treatment-related effects of chemotherapy, radiation, and anti-estrogen therapy, specific side effects/conditions that result in disrupted circadian rhythms, hospitalization, pain, and menopausal symptoms. *Perpetuating factors* in cancer patients include spending extended time in bed, taking frequent and long naps, following an irregular sleep schedule, and being physically inactive [16, 17]. In addition, beliefs such as fear of sleeplessness and worries about daytime consequences of poor sleep may delay sleep onset and cause frequent, prolonged awakenings. Decades of research into the cause of chronic insomnia have identified hyperarousal as a key factor, with increased levels of physiological, cognitive, and emotional levels of arousal in insomnia [15]. Hyperarousal, such as the hyper-activation of the stress system and pro-inflammatory cytokines, has been hypothesized to interact with unhelpful cognitive beliefs and negative behaviors contributing to insomnia perpetuation [15]. Insomnia may lead to depression by dysregulating the multiple systems involved in mood disorders [1], but in particular, it may favor a state of chonic inflammation hence contributing to depression. Insomnia is, in fact, associated with marked decreases in the numbers of T-cells and high levels of CRP and of both IL-6 and TNF [18].

Consequences of insomnia in cancer patients include irritable mood, psychological distress, daytime fatigue, increased pain symptomatology, increased chances of cancer recurrence, medication misuse and abuse, impaired cognitive functioning and quality of life [8-11]. The most likely hypothesis about the phenomenology of insomnia in cancer patients is that many stressors and challenges determined by the disease may contribute to insomnia, which in turn may exacerbate medical conditions comorbid with cancer, such as pain, psychiatric comorbidities, daytime fatigue, use of opioids (that contribute to daytime sedation), sleep-disordered breathing, misuse of stimulating or alerting drugs, and napping. Thus, a deleterious feedback loop may be created, and it may perpetuate not only insomnia but also cancer-related comorbid conditions such as depression, anxiety, symptoms of distress, as well as daytime fatigue and pain. Although the understanding of the causal relationship between insomnia/depression/fatigue in individuals with cancer is limited, these symptoms can exacerbate and maintain each other.

Therefore, interventions targeting insomnia could provide a promising approach for cancer-related symptoms among cancer patients. Cognitive Behavioral Therapy-for Insomnia (CBT-I) is the internationally considered first-line treatment for insomnia [19, 20], and it has been suggested as the preferred insomnia treatment in cancer patients and cancer survivors [12, 21, 22]. Besides improving insomnia symptoms, CBT-I in cancer patients has been shown to improve cancer-related symptomatology, such as daytime fatigues, anxiety, depressive and distress symptoms, overall quality of life, and indices of inflammation [23, 24]. CBT-Insomnia has been described effective during medication tapering and may be helpful to reduce hypnotics in cancer patients with insomnia [25]. CBT-I is a short intervention that usually consists of 4–8 weekly sessions and is usually delivered as a multi-component treatment and includes behavioral strategies such as psycho-education/sleep hygiene, relaxation training, stimulus control therapy, sleep restriction therapy, and cognitive strategies such as sleep/related cognitive restructuring [26]. CBT-I components often prove difficult for patients with cancer that can struggle even more from symptoms of fatigue. Thus, it has been suggested to incorporate in the usual CBT-I package an essential discussion of how to address these problems in cancer patients and to prepare strategies for reducing cancer-related fatigue providing patients with sleep education. Relaxation therapy has been shown a helpful intervention among cancer populations, with a possible secondary benefit on fatigue, distress, and indices of inflammation [21-24]. Exercise and mindfulness-based stress reduction and exercise interventions have been related to an improvement of sleep quality and daytime fatigue in cancer patients; hence it has been suggested to be useful for implementing the CBT-I approach in cancer patients [12]. If the CBT-I approach has failed for treating insomnia, international guidelines suggest the use of pharmacologic compounds for insomnia [19, 20, 27]. Previous guidelines on insomnia treatment in cancer patients have suggested applying indications valid for insomnia in the general population [8, 12]. Gamma-aminobutyric acid (GABAa) receptor agonists compounds such as short/ intermediate benzodiazepines (brotizolam, lormetazepam, temazepam, triazolam) and non-benzodiazepines compounds so-called Z-drugs including zolpidem, zaleplon, zopiclone, and ezopiclone are suggested for the treatment of insomnia in the short term (<4 weeks). Indeed, a variety of side-effects have been reported, including hangover, nocturnal confusion, falls, negative effects on next-day cognitive performance, rebound insomnia, tolerance, and dependency [20, 27]. Accordingly, they need to be used with caution, in particular in cancer patients which assume a lot of different therapies, Clinicians and patients should weigh the beneficial and harmful effects of medication according to individual circumstances, comorbidities, cancer type, stage of disease and/ or treatment characteristics. As a general rule, the dosage of the compounds should be kept to the minimum, and long-term use is advised against in cancer patients [8, 12]. Melatonin receptor agonists’ have been suggested as the first-line treatment for insomnia. Exogenous Melatonin 2 mg prolonged-release (PRM 2) is the compound that should be the preferred choice in subjects >55 years in light of its efficacy, and limited side-effects can be used within 13 weeks [20, 27]. PRM 2mg showed to mimics the physiological release of melatonin by releasing melatonin gradually and acting on melatonin receptors with chronobiotic effect. PRM 2 mg has been shown to be effective in improving sleep without altering the physiological sleep structure. PRM 2 mg has been shown to be well tolerated and not associated with impairment of psychomotor functions, memory recall, driving skills and have not shown side-effects such as hangover, nocturnal confusion and falls, negative effects on next-day cognitive performance, rebound insomnia, tolerance, and dependency [27]. In the last few years, it is emerging the role of melatonin as a safe and effective treatment for some forms of cancer, either alone or in combination with other therapies [28]. Due to its chronobiotic and its oncostatic properties, melatonin supplementation may be particularly useful in the treatment of insomnia in cancer [28].

In conclusion, insomnia is frequent in cancer because cancer patients are more predisposed to develop insomnia and are exposed to a myriad of precipitating factors. Dysfunctional behaviors about sleep may favor insomnia perpetuation, and a state of chronic inflammation with the hyperactivation of stress and pro-inflammatory systems may be established with insomnia. CBT-Insomnia should be the preferred insomnia treatment in cancer patients and cancer survivors and might be useful for improving not only insomnia symptoms but also cancer-related symptomatology, such as daytime fatigues, and depressive symptoms, overall quality of life, and indices of inflammation. Among pharmacological compounds, short/intermediate benzodiazepines and Z-drugs should be used with caution in cancer patients, which might assume a lot of different therapies, including opioid drugs while the use of Melatonin 2 mg prolonged-release may be particularly useful in the treatment of insomnia in cancer due to its chronobiotic and its oncostatic properties.

## 5 INSOMNIA AND CANCER RELATED FATIGUE

Cancer patients often describe cancer-related fatigue as more severe and more debilitating than ‘*normal*’ fatigue caused by lack of sleep and not relieved by adequate rest [29]. Fatigue may be debilitating before cancer treatments but typically increases during therapies, such as radiation, chemotherapy, hormonal, and/or biological therapies, with great variability in prevalence rates, ranging between 25 and 99%, due both to cancer type and treatments [30]. When severe, fatigue interferes with treatment continuation or treatment adherence. Studies on long-term survivors found that 25-30% of patients might experience persistent and disturbing levels of chronic fatigue up to 10 years after diagnosis [30].

Cancer-related fatigue usually manifests with physical, mental, and emotional signs and symptoms, including generalized weakness, diminished concentration or attention, decreased motivation or interest to engage in usual activities, and emotional lability, similar to that described by depressed patients. Considering its multi-dimensional characteristics, when protracted in the long-term, this syndrome has been postulated as a separate diagnostic entity and labeled as ‘*persistent cancer related fatigue*’ [31]. Cancer-related fatigue may be more adequately represented by a multi-factorial model that considers as relevant not only the biological factors but also the interpersonal and psychosocial ones. Other potential contributing factors include sleep disturbances and insomnia. In particular, insomnia has been identified as one of the seven factors that often influence cancer-related fatigue. Treatment of sleep disturbances is recommended in patients undergoing active cancer treatment, patients on long-term follow-up, and patients undergoing palliative and hospice care [10, 13]. A number of biological mechanisms have been investigated in order to give a rationale to several fatigue treatments, including cytokine dysregulations and hypothalamic-pituitary-adrenal axis dysregulations. Up to date, the most robust corpus of knowledge is on peripheral inflammatory cytokines and on the activation of the pro-inflammatory cytokine network as signaling central nervous system [32]. A number of inflammatory markers have been considered and investigated, namely the pro-inflammatory cytokines IL-1β, TNF-α, and IL-6 and markers of their activity, and CRP.

**Fig. (1):**
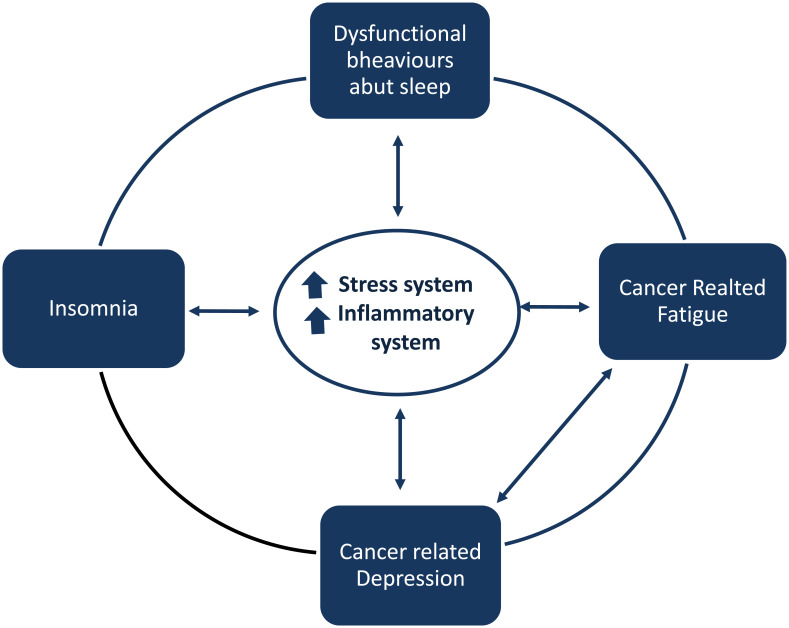
**Insomnia, fatigue, and depression: A self-reinforcing feedback loop in cancer** Insomnia in cancer is frequent, and it represents a factor contributing to both cancer-related depression and fatigue. When insomnia is established, it may lead to a vicious cycle with fatigue and depression and may, therefore, contribute to adverse cancer outcomes. Maladaptive behaviors and beliefs that patients feel may favor insomnia in cancer. These behaviors include spending extended time in bed, taking frequent and long naps, following an irregular sleep schedule, being physically inactive, use of opioids that contribute to daytime sedation, and misuse of stimulating or alerting drugs. Indeed, daytime fatigue, pain, and psychiatric comorbidities may contribute to insomnia. When insomnia is established, the hyper-activation of the stress system and proinflammatory cytokines are hypothesized to interact with unhelpful cognitive beliefs and negative behaviors contributing to insomnia perpetuation. Insomnia may favor cancer-related fatigue and depression and a deleterious feedback loop may be created. This feedback loop may perpetuate not only insomnia but also cancer-related comorbid conditions such as depression as well as daytime fatigue.

Studies on fatigue syndrome in long-term cancer survivors tried to determine what kind of mechanisms might be involved in the persistence of ‘*persistent cancer-related fatigue*’ even 5 to 10 years post-treatment. Consistent alterations in the pro-inflammatory cytokine network have been found among breast cancer, including elevations in circulating markers of inflammation [33], elevated intracellular cytokine production by monocytes [34], plasma levels of TNF receptor type II, especially among women treated with chemotherapy [35]. Dysregulated cortisol rhythm, reduced glucocorticoid receptor sensitivity, and alterations in the autonomic nervous systems have been investigated to explain the persistence of fatigue symptoms, even if the common pathway seems to be the close link between the dysregulation of these systems and their bi-directional relationships with inflammatory activity [36]. Insomnia and sleep disturbances have been hypothesized to enhance cancer-related fatigue through different mechanisms: they may contribute to psychological factors, such as depression and anxiety, to circadian sleep alterations, and to pain. Insomnia may also contribute to cancer-related fatigue by favoring cytokine and hypothalamic-pituitary-adrenal axis dysregulations (Fig. **1**). The treatment of insomnia with CBT- Insomnia has been shown to improve fatigue-related cancer [13].

In conclusion, cancer-related fatigue might have multi-factorial components, and these include insomnia and depression, which by establishing a state of chronic inflammation, may contribute to the pathogenesis of cancer-related fatigue. By treating insomnia with CBT-I, fatigue-related cancer may improve, and we may be able to interrupt the vicious cycle among insomnia, depression, and fatigue

## 6 INSOMNIA AND DEPRESSION IN CANCER

Around 30% of patients diagnosed with cancer may experience relevant levels of distress at some stage of their disease, with depression and anxiety as the most frequently diagnosed disorders three times higher than in the general population [37]. It is well known that psychiatric comorbidity and psychological distress related to cancer diagnosis/ treatments may negatively affect health outcomes, treatment adherence, overall quality of life, and immuno-regulation processes, thus affecting recovery rates [38, 39] with prolonged hospitalizations [40-42]. In a recent review of literature on quality of life in long-term survivors [43], pooled rates of 20-30% of individuals reporting ongoing physiological and psychological problems associated with cancer survivorship were found, thus highlighting the importance of a systematic assessment/treatment of psychiatric comorbidity or psychological distress. The definition of diagnostic boundaries for depression in cancer patients is also complicated by the presence, as already described, of fatigue and chronic fatigue with these entities as strongly correlated. Their relationships are bi-directional and rather simple, considering that fatigue can be a symptom of depression. Fatigue may also occur as a separate condition, and may interfere with social, occupational, interpersonal roles, thus enhancing the risk for a depressive syndrome. In fact, a number of longitudinal studies already demonstrated that subjects who experienced depression and anxiety symptoms might be more prone to develop a cancer-related fatigue syndrome in any stage of their clinical course [44-46]. Similarly, insomnia is widely recognized as a risk factor for depression; it may contribute to cancer-related depression, through different pathways [1]. Since depression has been related to the hyperactivation of immune-inflammatory signaling and the stress system, insomnia may contribute to the dysregulation of those systems involved in mood disorders [1, 18]. Hence, insomnia has been hypothesized to favor depression in cancer patients through the activation of the stress and inflammatory system in a combined effect with cancer its-self [38]. The treatment of insomnia with CBT-Insomnia has been shown to improve depression-related cancer and related indices of inflammation. In conclusion, cancer-related depression might have multifactorial components, and these may include insomnia, which by establishing a state of chronic inflammation, may contribute to the pathogenesis of depression. By treating insomnia with CBT-I, we may be able to address depressive symptoms in cancer as well, and we may be able to interrupt the vicious cycle among insomnia, depression, and fatigue.

## 7 THEORETICAL HYPOTHESES OF A SELF-REINFORCING FEEDBACK LOOP AMONG INSOMNIA, DEPRESSION, AND FATIGUE IN CANCER

Insomnia in cancer may favor cancer-related fatigue and cancer-related depression. When insomnia is established, it may lead to a vicious cycle with fatigue, depression and may account for adverse cancer outcomes. A deleterious feedback loop may be created, and it may perpetuate not only insomnia but also cancer-related comorbid conditions such as depression, as well as daytime fatigue. Although the understanding of the causal relationship between insomnia/ depression/fatigue in individuals with cancer is limited, these symptoms can exacerbate and maintain each other. We may hypothesize that cytokine and hypothalamic-pituitary-adrenal axis dysregulations related to insomnia may contribute to perpetuate this deleterious feedback loop in a combined effect with cancer itself (Fig. **1**). Since insomnia treatment has been shown to improve indices of inflammation in cancer with an improvement in fatigues, anxiety, depressive and distress symptoms, by targeting insomnia, we may be able to interrupt the vicious cycle among insomnia, fatigue, and depression.

## CONCLUSION

Insomnia in cancer is frequent, and it represents a factor contributing to both cancer-related depression and fatigue. When insomnia is established, it may lead to a vicious cycle with fatigue depression and may, contribute to adverse cancer outcomes. Thus, a deleterious feedback loop may be created, and it may perpetuate not only insomnia but also cancer-related comorbid conditions such as depression, anxiety, as well as daytime fatigue. Hence interventions targeting insomnia, in particular CBT-I insomnia treatment, have been shown to improve cancer-related symptomatology, such as daytime fatigues, anxiety, depressive and distress symptoms, overall quality of life, and indices of inflammation in cancer. Therefore, interventions targeting sleep disruption could provide a promising approach to cancer-related symptoms among cancer patients. By evaluating and addressing insomnia, we might improve mental/physical health and quality of life in cancer patients.
